# Increased Cortical Thickness in Attentional Networks in Parkinson's Disease with Minor Hallucinations

**DOI:** 10.1155/2019/5351749

**Published:** 2019-05-02

**Authors:** Caspian M. Sawczak, Alexander J. Barnett, Melanie Cohn

**Affiliations:** ^1^Department of Psychology, University of Toronto, 100 St. George Street, Toronto, ON, Canada M5S 3G3; ^2^Krembil Brain Institute at Toronto Western Hospital, 399 Bathurst Street, Toronto, ON, Canada M5T 2S8; ^3^Department of Psychology, University of California at Davis, 1 Shields Avenue, Davis, CA 95616, USA; ^4^Center for Neuroscience, University of California at Davis, 1544 Newton Court, Davis, CA 95618, USA

## Abstract

Hallucinations are common in Parkinson's disease (PD). Based on functional brain MRI data, hallucinations are proposed to result from alterations in the dorsal attention network (DAN), ventral attention network (VAN), and default mode network. Using structural MRI data from Parkinson's Progression Markers Initiative (PPMI), we examined cortical thickness in these networks in PD patients with (*n*=30) and without (*n*=30) minor hallucinations who were matched on multiple clinical characteristics (e.g., age, sex, education, cognitive diagnosis, MoCA score, medication, disease duration, and severity) as well as healthy controls (*n*=30) matched on demographic variables. Multivariate analyses revealed mild hallucinations to be associated with thicker cortex in the DAN and VAN, and these effects were driven by the left superior precentral sulcus and postcentral sulcus for the DAN and by the right insular gyrus for the VAN. While these findings may seem at odds with prior work showing grey matter reductions, our patients are in earlier stages of the disease than those in other studies. This is consistent with an inverted U-shape pattern of cortical thickness alterations in other neurodegenerative diseases and warrants further investigations in longitudinal studies tracking brain correlates of PD psychosis progression.

## 1. Introduction

Hallucinations are common in Parkinson's disease (PD) and follow a spectrum of severity (for review, see [1]). In their minor form, they consist of feeling someone's presence, passage hallucinations in one's peripheral vision, or increased frequency of visual illusions. With disease progression, well-formed visual hallucinations (e.g., people or animals) are experienced and insight is progressively lost leading to delusions. Importantly, hallucinations predict both dementia and nursing home placement in PD [2]. Neural correlates of PD-related hallucinations remain unclear. One model proposes that they develop due to dyscoordination of attentional brain networks in the context of ambiguous visual percepts [3, 4]. Specifically, alterations in the goal-directed, top-down, dorsal attention network (DAN) render it unable to correct erroneous interpretations of ambiguous visual stimuli by the stimulus-driven, bottom-up, ventral attention network (VAN) [5] and the default mode network (DMN) which mediates internally generated thoughts [6]. Normally, activity in the DAN and VAN are coupled and are anticorrelated with that of the DMN, but in PD with hallucinations, these relationships are altered. DAN activity and its connectivity with the VAN and visual regions are reduced, while connectivity within and between the DMN and VAN is increased [4].

To date, alterations in these networks are partially supported by structural neuroimaging findings. PD-related hallucinations have been associated with reduced grey matter volume in regions of the DAN (e.g., superior and inferior parietal lobules), the VAN (e.g., right insula), and DMN (e.g., hippocampus) [7], as well as atrophy in various components of the visual pathway [8, 9]. However, these findings are inconsistent across studies possibly due to small samples and variability in methodologies. Most structural MRI studies also investigate PD patients with well-formed visual hallucinations at relatively late stages of the disease. One exception is a study demonstrating cortical thinning in the supramarginal, superior frontal, and lateral occipital gyri in a small patient group with well-formed visual hallucinations early in the disease course, but showing no alterations in patients with minor hallucinations (MH) [2]. Interestingly, although these findings were not interpreted in the context of the attentional network dysregulation model, the three regions showing cortical thinning are part of the DAN, VAN, and DMN [10]. In contrast, although hippocampal atrophy was found in advanced PD with well-formed hallucinations [11], increased volume was found in patients with MH [12]. In the present study, we address these conflicting results by investigating cortical thickness alterations in the VAN, DAN, and DMN in recently diagnosed PD patients with MH (PDMH) compared to PD patients with no hallucinations (PDnH), who were matched on multiple demographic and clinical variables, and healthy controls (HC). Based on the attentional network dysfunction model, we hypothesized that the PDMH group would demonstrate cortical thickness alterations in these networks.

## 2. Methods

### 2.1. Participants

Data used in the preparation of this article were obtained from Parkinson's Progression Markers Initiative (PPMI) database (http://www.ppmi-info.org/data). For up-to-date information on the study, visit http://www.ppmi-info.org. Each participating PPMI site received approval from an ethical standards committee on human experimentation before study initiation and obtained written informed consent for research from all individuals participating in the study. We extracted T1-weighted structural MRI, along with clinical and demographic data for 90 individuals (30 HC; 30 PDnH; 30 PDMH). The presence of MH was defined by a score of “1” on question 1.2 of the MDS-UPDRS on at least three consecutive visits (each at 6-month intervals). This, combined with review of participants' medical history confirming the absence of schizophrenia-spectrum disorder and of current dementia, is consistent with diagnostic criteria for PD-related psychosis, excluding dementia with Lewy bodies [13, 14]. Conversely, the PDnH patients reported no hallucinations on all visits to date. The groups were matched, participant-by-participant for age, sex, education, PD severity (MDS-UPDRS Part III and Hoehn and Yahr), whether they took PD medications, and cognitive diagnosis (i.e., mild cognitive impairment (MCI) versus intact, determined by PPMI investigators based on clinical interviews and cognitive testing; neuropsychological data are presented in Supplementary Materials (available here)). PD groups were also matched for levodopa-equivalent daily dose (LEDD) and MoCA scores. Demographic and clinical characteristics are shown in Table 1. For the PDMH group, MRI scans were selected from visits at the closest time point from their MDS-UPDRS. Scans for other groups were selected based on participant age, matched to the PDMH group.

### 2.2. Preprocessing and Cortical Thickness Measurements

T1-weighted structural MRI scans were acquired on 3T scanners using Sagittal 3D MP-RAGE or 3D FSPGR with an approximate 1 mm isotropic voxel size (see ppmi.org for details on cross-sites quality control and standardization). Cortical reconstruction and volumetric segmentation were performed using FreeSurfer-v4.5 (http://surfer.nmr.mgh.harvard.edu) [15]. Preprocessing included intensity normalization, skull stripping, registration to standard space, smoothing, delineation of pial, white matter, and grey matter surfaces with manual corrections, and parcellation of the cortex into 150 regions of interest (ROIs). We extracted each participant's cortical thickness for 31 ROIs defined by matching FreeSurfer parcellations [15] to peak MNI coordinates of key nodes of the DAN, DMN, and VAN identified by Yeo et al. [10]. Following visual inspection, we added two FreeSurfer parcellations to the DMN and removed one from the VAN to improve the spatial extent overlap between our network coverage and that of Yeo et al. (Supplementary Materials (available here) for network renderings, MNI coordinates, and corresponding FreeSurfer labels). We used bilateral ROIs for the DAN (superior precentral sulcus, posterior sulcus, superior parietal lobule, and anterior occipital sulcus) and DMN (superior frontal sulcus, angular gyrus, middle temporal gyrus, anterior cingulate cortex, superior frontal gyrus, parahippocampal gyrus, dorsal posterior cingulate cortex, and precuneus), and right hemisphere ROIs for the VAN (supramarginal gyrus, planum temporale, anterior middle cingulate, margin of the cingulate sulcus, and short insular gyrus), as it is known to be right-lateralized [5, 16]. Estimates of hippocampal volume were also extracted, given that this subcortical structure is part of the DMN and showed mixed findings in PD with hallucinations [1, 7].

## 3. Statistical Analysis and Results

### 3.1. Demographics and Clinical Measures

Using ANOVAs to compare the three groups (Table 1), there were no significant differences in age (*F*_(2,89)_ = 0.084, *p*=0.919) or education (*F*_(2,89)_ = 0.688, *p*=0.505) but the groups differed on the MoCA (*F*_(2,89)_ = 9.221, *p* < 0.001); post hoc tests (with Bonferroni corrections) revealed that HC performed better than the PDMH (*p* < 0.001) and PDnH (*p*=0.02) groups, but the two PD groups did not differ (*p*=0.455). Within PD, *t*-tests revealed that the PDMH and PDnH groups did not differ significantly on LEDD (*t*_(27)_ = 0.424, *p*=0.675), disease duration (*t*_(58)_ = 1.702, *p*=0.094), or disease severity as measured by the MDS-UPDRS Part III (*t*_(58)_ = 1.685, *p*=0.097). Similarly, a median test showed no significant difference between PD groups on Hoehn and Yahr (*p*=0.347). Chi-squared tests showed no differences between PD groups in medication types (Levodopa: *χ* ^2^ = 1.926, *p*=0.165; DA agonists, *χ* ^2^ = 2.308, *p*=0.129; other PD medications, *χ* ^2^ = 1.002, *p*=0.317; antipsychotics, *χ* ^2^ = 2.069, *p*=0.15), presence of dyskinesia (*χ* ^2^ = 1.017, *p*=0.313), or presence of REM sleep behaviour disorder (*χ* ^2^ = 2.500, *p*=0.114).

### 3.2. Cortical Thickness

To correct cortical thickness measurements, we regressed out age, sex, and a proxy measure of brain volume (pBV = white matter + subcortical grey matter + cerebrospinal fluid) and the resulting standardized residuals were used in our primary analysis. The pBV excluded cortical grey matter to avoid controlling for the measure we are interested in (cortical thickness). The corrected standardized residuals were used as the dependent variables in mean-centered task partial least square (PLS) analyses [17] (see [17] for PLS review and tutorial). PLS is a multivariate technique that analyzes the covariance between a design matrix (i.e., group membership) and a data matrix (i.e., cortical thickness residuals). We performed separate analyses for the DAN, VAN, and DMN. A matrix of correlations between these two matrices was computed and used as the input for a singular value decomposition, which identifies latent variables that best explain the variance in the data. *p* values for the latent variables are obtained via comparison to a null distribution generated by randomly permuting the data matrix 1000 times and computing new correlations with the design matrix each time. Next, 500 bootstraps were performed by randomly resampling participants with replacement, which allowed calculation of the standard error of the salience of each ROI within the latent variables. As this is done in a single step in this multivariate technique, correction for multiple comparisons is not necessary.

**Figure 1:**
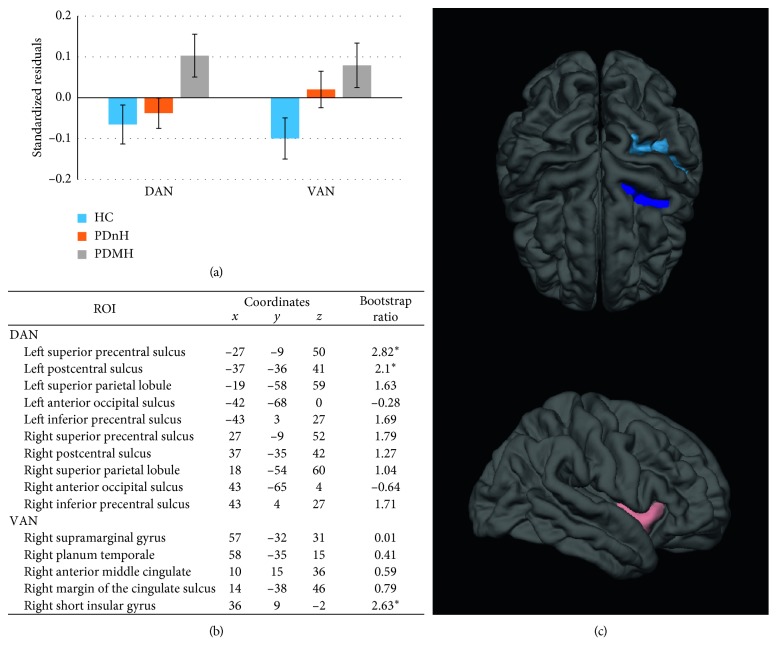
Group difference in cortical thickness. (a) Linear contrasts for each PLS network analysis. (b) Central MNI coordinates of the FreeSurfer parcellations and bootstrap ratios (BSRs) for all ROIs within the DAN and VAN. Asterisks indicate the most salient ROIs (BSR ≥ 2) contributing to each pattern. (c) Most salient ROIs in the DAN (dark blue = left frontal eye fields; light blue = left inferior parietal lobule) and VAN (orange = right insula). DMN is not depicted as no significant latent variable was found.

Significant latent variables were identified for the DAN (*p*=0.039, explained covariance = 75.5%) and the VAN (*p*=0.027, explained covariance = 69.7%), but no significant latent variable was identified for the DMN. Linear contrasts revealed that cortical thickness was greater in the DAN among PDMH patients compared to the other two groups and thicker in the VAN among PDMH compared to HC. Bootstrap ratios (BSRs) and MNI coordinates for the ROIs in each network are shown in Figure 1. BSRs are roughly proportional to z-scores but should be interpreted as confidence intervals. In the present study, we consider BSRs ≥ 1.96 to be statistically significant; this corresponds to a 95% confidence interval. The most salient ROIs contributing to the group differences in the DAN were, as defined by the FreeSurfer Atlas [15], the left superior precentral sulcus and left postcentral sulcus (BSRs = 2.82 and 2.1, respectively) and in the VAN, the right short insular gyrus (BSR = 2.63). The peak MNI coordinates of these FreeSurfer ROIs correspond to canonical DAN and VAN areas identified in Fox et al. [18] seminal paper [18] as the frontal eye fields, inferior parietal lobule, and insula, respectively. We also compared groups' hippocampal volumes including age, sex, and intracranial volume as covariates of no interest in ANCOVAs. There was no effect of group in left (*F*_(2,89)_ = 1.831, *p*=0.167) nor right hippocampal volume (*F*_(2,89)_ = 0.429, *p*=0.653).

## 4. Discussion

Our data partially support our predictions that individuals with early PD and MH exhibit cortical thickness alterations in attentional networks. Specifically, we found that the PDMH group had thicker cortex in the DAN compared to PDnH and HC groups, and this effect was driven by the left superior precentral sulcus and postcentral sulcus. We also found the PDMH group to have thicker cortex in the VAN compared to HCs, and this effect was driven by the right short insular gyrus. We did not find any group differences in cortical thickness in the DMN cortical ROIs nor in hippocampal volume. Others have found PD hallucinations to be associated with either reduced [2, 11] or increased [12] hippocampal volume; these mixed findings may be attributable to differences in hallucination duration or severity, as we will discuss shortly.

The presence of alterations in the VAN and DAN is consistent with the attentional dysfunction model of PD hallucinations [3, 4]. Our results of increased rather than decreased cortical thickness may seem at odds with studies showing reduced grey matter volume and cortical thinning in some brain areas affiliated with the DAN and VAN [2, 7]. However, similar findings of increased thickness/volume have been documented in PD patients with MH in the hippocampus and in other regions such as the parahippocampal gyrus and orbitofrontal cortex [12].

First, several methodological differences may contribute to these discrepancies. We used a multivariate technique and an ROI approach, which may allow detection of more subtle effects than whole-brain univariate analyses. We also used cortical thickness analyses, whereas most previous studies used VBM which is less specific in that it conveys information about a combination of grey matter measures (surface area, cortical folding, and cortical thickness) [19]. Importantly, our PD sample has a short disease duration with only MH while other studies involve PD with longer disease duration and well-formed visual hallucinations. Thus, it is possible that thicker cortex manifests early in the disease before giving way to atrophic processes, following an inverted U-shape function. A similar rationale was provided to explain increased hippocampal and cerebellar volumes in PD patients with MH [12]. Such an inverted U-shape function is seen in other conditions such as prodromal Alzheimer's disease (AD). For instance, temporal and parietal structural changes in presenilin-1 mutation carriers follow a nonlinear trajectory with regional increases in presymptomatic period followed by decreases over time [20, 21]. We argue this inverted U-shape model potentially can explain the discrepancy between the present and previous work showing reduced, rather than increased, grey matter in the right insula in PD hallucinations [2]. The average disease duration of Shine et al.'s [3] PD hallucination sample was 6.7 years, which is approximately three times that of our PDMH sample. If grey matter alterations in PD hallucinations do indeed follow an inverted U-shape trajectory as the pathology progresses, then our finding and Shine et al.'s can be reconciled. Additionally, this increased cortical thickness in the VAN and DAN may reflect a trait or risk factor for the development of hallucinations. Interestingly, increased cortical thickness in different brain regions including the insula and inferior parietal lobule has been reported in individuals with schizophrenia who experience auditory hallucinations [22], which was interpreted as a dysregulation in cortical development. Similarly, increased cortical thickness in the superior parietal lobule has also been documented in patients with bipolar disorders with a lifetime history of auditory hallucinations [23].

One limitation of the present study is the reliance on a single item from the MDS-UPDRS scale (item 1.2) to determine the presence or absence of MH. Although the requirement of three consecutive (spaced at 6-month intervals) negative ratings in the PDnH and positive ratings in the PDMH minimizes possible false-negative and false-positive classification errors, respectively, this item does not provide detailed information on the qualitative nature and diversity of the minor hallucinations experienced. This is a common criticism of studies on PD-related psychosis. As a result, recently developed structured interviews have been validated and used in PD research to characterize hallucinations and their related features in more details. Some of these instruments only focus on well-formed hallucinations (e.g., North-East Visual Hallucinations Interview (NEVH I) [24] and Scale for Assessment of Positive Symptoms adapted for PD (SAPS PD) [25]), but a recent adaptation also surveys minor hallucinations (e.g., enhanced SAPS PD (eSAPS-PD) [26]). Although the PPMI study does not include such instruments, their integration in future research will certainly enhance our understanding of hallucinations in PD and underlying neural substrates.

## 5. Conclusion

In sum, we provide evidence for cortical thickness alterations in attention networks in recently diagnosed PD patients experiencing MH, which supports the attentional dysfunction model of PD hallucinations. Our data also suggest that the course of cortical changes may not follow a linear trajectory but rather an inverted U-shape function, as in prodromal AD, and future work should involve within-subject longitudinal investigations of the progression of psychosis to test this notion.

## Figures and Tables

**Table 1:** Demographic and clinical characteristics.

	HC (*N* = 30)		PDnH (*N* = 30)		PDMH (*N* = 30)		*p* value	Post hoc
Demographics								
Age (years)	63.2	(9.5)	62.7	(8.1)	63.7	(9.7)	0.919	
Male, *n* (%)	19	(63)	19	(63)	19	(63)		
Education (years)	16.0	(2.9)	15.1	(3.1)	15.6	(2.9)	0.505	

Clinical								
Years since diagnosis	—	—	1.7	(1.1)	2.3	(1.4)	0.094	
MDS-UPDRS Part III	—	—	21.0	(10)	25.7	(11.3)	0.097	
Hoehn and Yahr, median (range)	—	—	2	(1–3)	2	(1–3)	0.347	
Dyskinesia present, *n* (%)	—	—	1	(3)	0	(0)	0.313	
REM sleep behaviour disorder, *n* (%)	—	—	9	(30)	15	(50)	0.114	
Medication for PD, *n* (%)	—	—	12	(41)	17	(57)	0.240	
Levodopa, *n* (%)	—	—	7	(23)	12	(40)	0.165	
DA agonist, *n* (%)	—	—	2	(7)	6	(20)	0.129	
Other, *n* (%)	—	—	4	(13)	7	(23)	0.317	
LEDD (mg)	—	—	370.2	(225.5)	410.9	(273.9)	0.675	
Antipsychotic medication (quetiapine), *n* (%)	—	—	0	(0)	1^a^	(3)	0.313	
Duration of hallucinations (years)	—	—	—	—	1.23	(0.66)	—	

Cognition								
Cognitive state (intact: MCI)	30 : 0		20 : 10		20 : 10		—	
MoCA	28.1	(1.8)	26.5	(2.3)	25.7	(2.5)	<0.001	HC > PDnVH and PDMH

Notes: demographic, clinical, and cognitive characteristics of our sample. Mean and standard deviation (parentheses) except where otherwise noted. ANOVAs were conducted for each measure and, if significant (*p* ≤ 0.05), group differences were ascertained through post hoc testing. DA = dopamine; LEDD = levodopa equivalent daily dosage; MCI = mild cognitive impairment; MoCA = Montreal Cognitive Assessment. ^a^Quetiapine 25 mg initiated 6 years prior to the scan date to treat anxiety.

## Data Availability

All data were collected by Parkinson's Progression Markers Initiative (PPMI) and are available, upon request, online (https://www.ppmi-info.org/access-data-specimens).

## Supplementary Materials

Supplementary Materials The Supplementary Materials contain additional data on neuropsychological assessments, as well as a detailed description of how structural brain networks and ROIs were identified.
