# Supplementary material for: Increased Cortical Thickness in Attentional Networks in Parkinson's Disease with Minor Hallucinations
Source: Parkinsons Dis. 2019 May 2;2019:5351749. doi: 10.1155/2019/5351749 (PMC6525808; doi:10.1155/2019/5351749)
Supplement: Supplementary Materials — The Supplementary Materials contain additional data on neuropsychological assessments, as well as a detailed description of how structural brain networks and ROIs were identified. [file 5351749.f1.pdf]

Supplementary Materials 1

Cognitive Tests

While participants' performance on the Montreal Cognitive Assessment (MoCA; Nasreddine et al., 2005), a general screening instrument, is reported in the manuscript, we recognize that some readers may be interested in more detailed cognitive characterization of our sample. Group scores for each neuropsychological test used as part of the PPMI study to support PD-MCI diagnosis are provided in Table S1. Description of each measures can be found in [1].

For each test, we ran an ANOVA comparing Parkinson's patients with minor hallucinations (PDMH), Parkinson's patients with no hallucinations (PDnH), and healthy controls (HC), and when significant, we ran post-hoc tests (Bonferroni-corrected). We found no significant effect of group ($F_{(2,89)}$=1.183, p=.311) on the Benton Judgment of Line Orientation test (BJLOT), which measures visuospatial skills. There was also no significant effect of group in memory function as measured by the Hopkins Verbal Learning Test (HVLT) total free recall ($F_{(2,89)}$=1.214, p=.302), or recognition discriminability index ($F_{(2,89)}$=.146, p=.864), as well as no significant group effect on an animal semantic verbal fluency task measuring executive functioning and language ($F_{(2,89)}$=2.486, p=.089).

In contrast, significant group effects were found on measures of working memory and attention/processing speed. Specifically, group differences were noted on the Letter Number Sequencing subtest of the Wechsler Adult Intelligence Scale III (WAIS-III) ($F_{(2,89)}$=3.823, p=.026) and the Symbol Digit Modalities test (SDMT) ($F_{(2,89)}$=4.063, p=.021), and post-hoc tests (Bonferroni-corrected) indicated that both were due to HCs scoring significantly higher than PDMHs on both measures (p=.032 and p=.018, respectively). However, there was no significant difference between HC and PDnH (p=.117; p=.892), and critically, between PDMH and PDnH (p≈1; p=.240). The noted difference from HC are not surprising given that unlike PD groups, no HC participants have MCI.

Table S1. Cognitive test scores

	HC (N=30)		PDnH (N=30)		PDMH (N=30)		p-value	Post-hoc
BJLOT	20.8	(8.0)	23.0	(6.4)	20.4	(7.1)	.311	-
Semantic fluency	22.8	(5.7)	20.7	(4.0)	19.9	(6.0)	.089	-
Letter-Number Sequencing	11.6	(3.0)	10.1	(2.9)	9.8	(2.4)	.026	HC >PDMH
Symbol Digit Modalities	46.0	(13.2)	42.7	(9.1)	37.0	(14.0)	.021	HC >PDMH
HVLT, total recall	26.4	(3.7)	24.6	(5.1)	24.8	(5.6)	.302	-
HVLT, discrimination index	10.0	(2.8)	9.7	(2.9)	9.7	(3.0)	.864	-

Table 1. Mean and standard deviation (parentheses) of each group's performance on cognitive tests. ANOVAs were conducted for each measure and, if significant ($p \leq .05$), group differences were ascertained through post-hoc testing. BJLOT = Benton Judgment of Line Orientation Test; HVLT = Hopkins Verbal Learning Test.

References

1. Strauss, E., E.M. Sherman, and O. Spreen, *A compendium of neuropsychological tests*. 2006, New York: Oxford University Press.

Supplementary Materials 2

Mapping of structural networks and selection of ROIs

FreeSurfer-v4.5 (http://surfer.nmr.mgh.harvard.edu; [1]) was used to segment each subject's cortex into 150 regions of interest (ROIs), according to FreeSurfer's Destrieux atlas [2]. Next, we selected ROIs corresponding to peak regions in networks identified by Yeo et al. [3] by matching peak MNI coordinates as closely as possible for each ROI (see Table S2). We then rendered the networks resulting from this process on a default brain in FreeSurfer and visually compared them to renderings of Yeo et al.'s [3] networks (see Figure S1), which are available online (https://surfer.nmr.mgh.harvard.edu/fswiki/CorticalParcellation_Yeo2011). Doing so revealed some small discrepancies in the extent of cortical surface area covered in the default mode network (DMN) and ventral attention network (VAN). To rectify these discrepancies and maximize the overlap between our networks and those of Yeo et al. [3], we selected two additional FreeSurfer ROIs for the DMN and excluded one from the VAN. Specifically, we added the superior frontal gyrus and precuneus to the DMN to increase the extent of coverage. We also excluded the middle frontal gyrus from the VAN as the corresponding FreeSurfer ROI has a much broader extent encompassing on brain areas belonging to the DMN in Yeo's network. Last, since Yeo et al. [3] only identify ROIs and list their MNI coordinates in the left hemisphere, our mapping was done using FreeSurfer's left hemisphere ROIs. We then added FreeSurfer's right hemisphere counterparts to the DAN and DMN, and for the VAN we used only the right hemisphere ROIs (see Table S2), since that network is known to be right-lateralized [4, 5].

Table S2. Labels and coordinates of bilateral or right hemisphere FreeSurfer ROIs corresponding to left ROIs identified by Yeo et al. (2011).

Yeo et al. (2011)				FreeSurfer (Destrieux atlas)				
ROI	MNI coordinates			Label	MNI coordinates			Long name
	x	*y*	*z*		*x*	*y*	*z*	
Dorsal attention A	-22	-8	54	S_precentral-sup-part	-/+27	-9	50	Superior precentral sulcus
Dorsal attention B	-34	-38	44	S_postcentral	-/+37	-36	41	Postcentral sulcus
Dorsal attention C	-18	-69	51	G_parietal_sup	-/+19	-58	59	Superior parietal lobule
Dorsal attention D	-51	-64	-2	S_occipital_ant	-/+42	-68	0	Anterior occipital sulcus
Dorsal attention E	-8	-63	57	G_parietal_sup	-/+19	-58	59	Superior parietal lobule
Dorsal attention F	-49	3	34	S_precentral-inf-part	-/+43	3	27	Inferior precentral sulcus
Default A	-27	23	48	S_front_sup	-/+23	18	43	Superior frontal sulcus
Default B	-41	-60	29	G_pariet_inf-Angular	-/+43	-64	38	Angular gyrus
Default C	-64	-20	-9	G_temporal_middle	-/+59	-36	-13	Middle temporal gyrus
Default D	-7	49	18	G_and_S_cingul-Ant	-/+11	39	8	Anterior cingulate cortex
Default D	-7	49	18	G_front_sup	-/+9	22	51	Superior frontal gyrus
Default E	-25	-32	-18	G_oc-temp_med-Parahip	-/+24	-19	-27	Parahippocampal gyrus
Default F	-7	-52	26	G_cingul-Post-dorsal	-/+5	-36	31	Dorsal posterior cingulate cortex
Default F	-7	-52	26	G_precuneus	-/+8	-59	45	Precuneus
Ventral attention A	-31	39	30	NA	NA	NA	NA	NA
Ventral attention B	-54	-36	27	G_pariet_inf-Supramar	56	-36	32	Supramarginal gyrus
Ventral attention C	-60	-59	11	G_temp_sup-Plan_tempo	57	-41	15	Planum temporale
Ventral attention D	-5	15	32	G_and_S_cingul-Mid-Ant	10	14	36	Anterior middle cingulate cortex
Ventral attention E	-8	-24	39	S_cingul-Marginalis	15	-38	45	Marginal branch of the cingulate sulcus
Ventral attention F	-31	11	8	G_insular_short	35	9	-3	Short insular gyrus

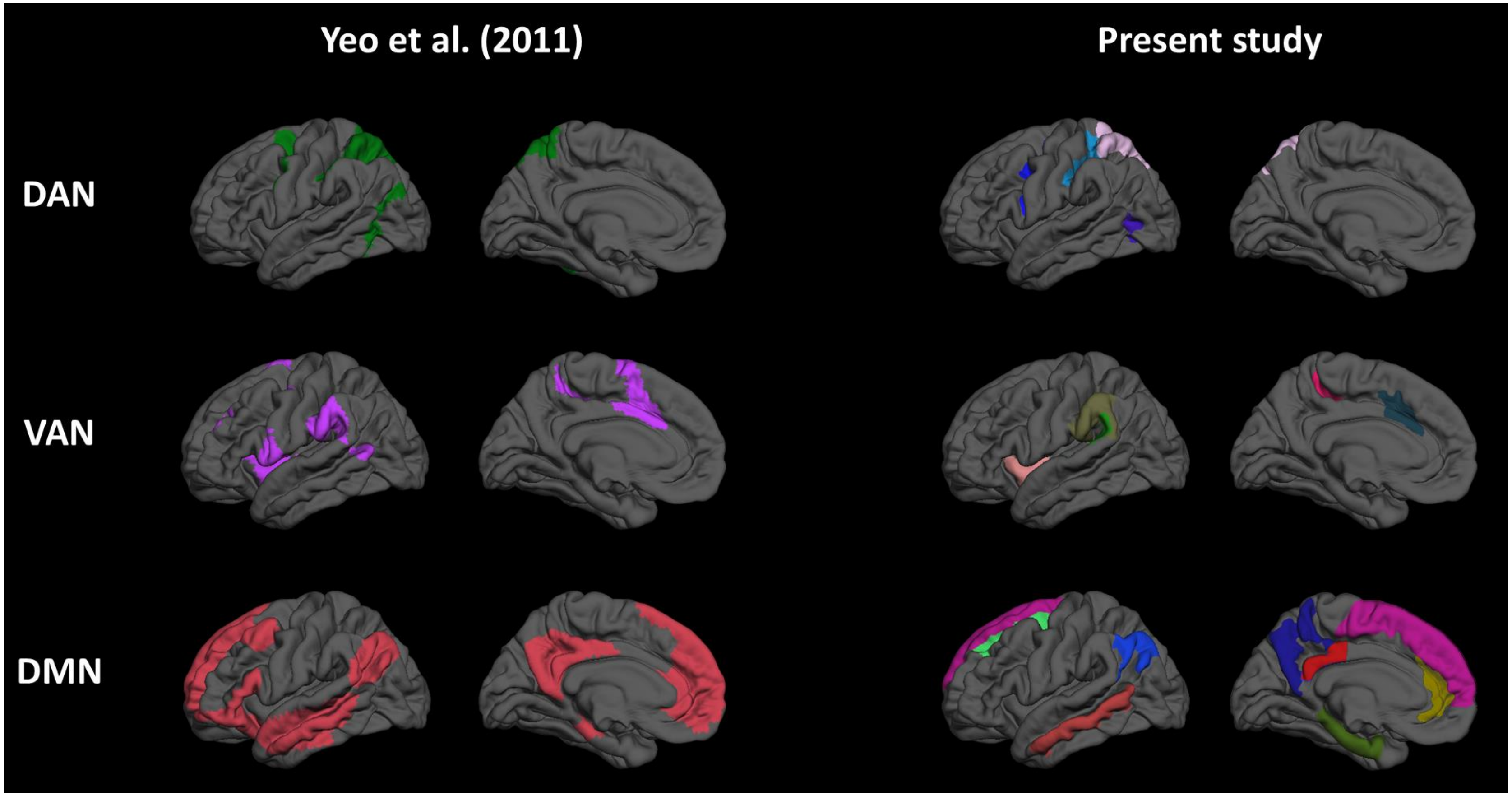

Figure S1. Left panel: Networks as defined by Yeo et al. (2011). Right panel: The present study's approximation of Yeo et al.'s (2011) network coverage, accomplished by matching FreeSurfer ROIs as closely as possible by MNI coordinates. DAN = dorsal attention network; VAN = ventral attention network; DMN = default mode network. FreeSurfer ROI legend: DAN, dark blue = inferior and superior precentral sulci, light blue = postcentral sulcus, light pink = superior parietal lobule, purple = anterior occipital sulcus; VAN, salmon = short insular gyrus, olive = supramarginal gyrus, green = planum temporale, magenta = marginal branch of the cingulate sulcus, teal = anterior middle cingulate cortex; DMN, pink = superior frontal gyrus, light green = superior frontal sulcus, orange = middle temporal gyrus, blue = angular gyrus, indigo = precuneus, yellow = anterior cingulate cortex, red = dorsal posterior cingulate cortex, dark green = parahippocampal gyrus.

Supplementary references

1. Fischl, B., M.I. Sereno, and A.M. Dale, *Cortical surface-based analysis. II: Inflation, flattening, and a surface-based coordinate system.* Neuroimage, 1999. **9**(2): p. 195-207.
2. Destrieux, C., et al., *Automatic parcellation of human cortical gyri and sulci using standard anatomical nomenclature.* Neuroimage, 2010. **53**(1): p. 1-15.
3. Yeo, B.T., et al., *The organization of the human cerebral cortex estimated by intrinsic functional connectivity.* J Neurophysiol, 2011. **106**(3): p. 1125-65.
4. Corbetta, M. and G.L. Shulman, *Control of goal-directed and stimulus-driven attention in the brain.* Nat Rev Neurosci, 2002. **3**(3): p. 201-15.
5. Vossel, S., J.J. Geng, and G.R. Fink, *Dorsal and ventral attention systems: distinct neural circuits but collaborative roles.* Neuroscientist, 2014. **20**(2): p. 150-9.
